# Impact of Epigenetics on Complications of Fanconi Anemia: The Role of Vitamin D-Modulated Immunity

**DOI:** 10.3390/nu12051355

**Published:** 2020-05-09

**Authors:** Eunike Velleuer, Carsten Carlberg

**Affiliations:** 1 Children’s Hospital Neuwerk, D-41066 Mönchengladbach, Germany; e.velleuer@kh-neuwerk.de; 2 Department for Cytopathology, Heinrich-Heine-University Düsseldorf, D-40225 Düsseldorf, Germany; 3 Institute of Biomedicine, University of Eastern Finland, FI-70211 Kuopio, Finland

**Keywords:** vitamin D, Fanconi anemia, epigenetics, cancer, immunology, prevention

## Abstract

Fanconi anemia (FA) is a rare disorder with the clinical characteristics of (i) specific malformations at birth, (ii) progressive bone marrow failure already during early childhood and (iii) dramatically increased risk of developing cancer in early age, such as acute myeloid leukemia and squamous cell carcinoma. Patients with FA show DNA fragility due to a defect in the DNA repair machinery based on predominately recessive mutations in 23 genes. Interestingly, patients originating from the same family and sharing an identical mutation, frequently show significant differences in their clinical presentation. This implies that epigenetics plays an important role in the manifestation of the disease. The biologically active form of vitamin D, 1α,25-dihydroxyvitamin D_3_ controls cellular growth, differentiation and apoptosis via the modulation of the immune system. The nuclear hormone activates the transcription factor vitamin D receptor that affects, via fine-tuning of the epigenome, the transcription of >1000 human genes. In this review, we discuss that changes in the epigenome, in particular in immune cells, may be central for the clinical manifestation of FA. These epigenetic changes can be modulated by vitamin D suggesting that the individual FA patient’s vitamin D status and responsiveness are of critical importance for disease progression.

## 1. Introduction

**Figure 1:**
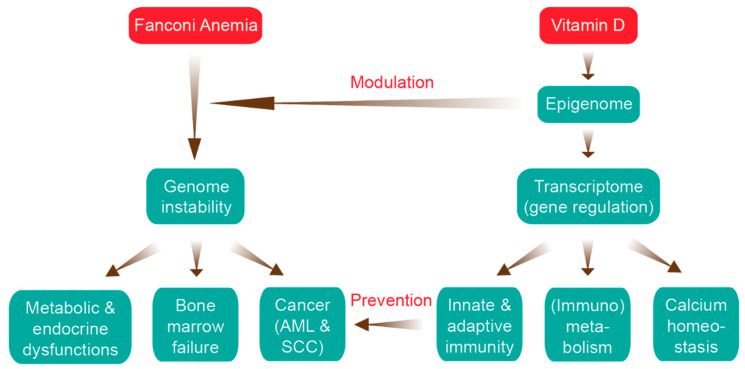
Relation of Fanconi anemia (FA) and vitamin D. The major clinical features of FA (left) are aligned with the key functions of the vitamin D system (right, more details in Figures 3 and 4). There are two main ways that vitamin D can interfere with the FA disease progression: epigenetic modulation of genome instability and prevention of cancer onset via activation of the immune system. AML, acute myeloid leukemia; SCC, squamous cell carcinoma.

Fanconi anemia (FA) is a rare (1:300,000) inherited disease that is one of the leading causes of bone marrow failure and inherited bone marrow failure syndromes [1,2]. FA results from defects in the FA/breast cancer gene DNA repair pathway leading to chromosomal fragility [3]. FA confers with a very high predisposition to myelodysplastic syndrome and cancers, such as acute myeloid leukemia and squamous cell carcinoma [4] (Figure 1). In general, the genetic basis of cancer is an accumulation of point mutations, amplifications and deletions as well as big chromosomal alterations like translocations and aneuploidies that by different mechanisms enhance the activity of oncogenes or decrease the actions of tumor suppressor genes [5,6]. However, most non-communicable diseases, such as cancer, have a not only a genetic, inherited basis but also an epigenetic component.

Epigenetics covers all changes of the genome that are functionally relevant but do not involve any alteration in the nucleotide sequence [7]. This includes the cell- and tissue-specific packaging and accessibility of genomic DNA [8], which includes covalent and structural modifications of chromatin, such as cytosine methylation, post-translational modifications of histones and the 3-dimensional organization of the nucleus [9]. Epigenomics, the genome-wide view on epigenetics, is of key importance, when during embryogenesis, i.e., during the first weeks of life, embryonic stem cells differentiate into specialized cell types [10]. Moreover, epigenetics plays also a central role when adult stem cells in bone marrow, intestine or skin grow and differentiate, in order to replace dead cells in the immune system or the inner and outer surface of the body [11]. Cancer cells contain epimutations, which are changes in DNA methylation, histone modifications and chromatin structure [12]. Accordingly, tumorigenesis is associated with major epigenetic changes like in embryogenesis and often the respective epigenomic program of early development is activated [13]. Mechanistically, cancer epigenetics is based on metabolic and environmental stimuli disrupting the homeostatic balance of chromatin [14]. Thus, epigenetics plays a central role in cancer, both in prevention and in therapy.

In contrast to the static genome, the epigenome is very dynamic and responds to intra- and extracellular signal transduction cascades, which are induced, e.g., by peptide hormones, cytokines and growth factors [15]. Importantly, the most dominant external signals are dietary compounds, such as polyunsaturated fatty acids and lipophilic hormones like 1α,25-dihydroxyvitamin D_3_ (1,25(OH)_2_D_3_), that directly activate transcription factors like peroxisome proliferator-activated receptors (PPARs) or vitamin D receptor (VDR), respectively. In addition, the signals can work via intermediary metabolites, such as α-ketoglutarate, being an essential co-substrate of chromatin modifying enzymes like lysine demethylases and DNA demethylating TET proteins [16]. Both types of nuclear proteins induce changes to the epigenome, such as increased chromatin accessibility as well as demethylation of histones and genomic DNA. This finally results in alterations in the expression of protein-coding and non-coding RNA genes. Some of these changes are transient, while others leave persistent marks on the epigenome, i.e., they can memorize environmental events, such as food, infection with microbes or stress. Thus, epigenomics also explains how the genome is connected with the environment [17]. This highlights the interesting perspective that dietary choices for macronutrients, such as polyunsaturated fatty acids, and micronutrients, such as vitamin D_3_, can prevent or at least delay the onset of cancer and other disorders [18,19].

Although, in principle, the genome can be edited by methods like CRISPR-Cas9, efficient genome editing is very difficult to achieve for the trillions of cells of an adult body and may lead to undesired off-target effects, i.e., genome changes at sites other than the targeted ones [20]. In contrast, there are many safe ways to change the epigenome, which are primarily based on lifestyle choices and environmental exposures [21]. This suggests that the genetic predisposition for a disease, such as FA, may be balanced through a lifestyle change affecting the epigenome. Interestingly, patients with FA differ significantly in their clinical presentation, even if they originate from the same family, i.e., they share the identical mutation [22,23]. In the cancer field, it is well known that genomic instabilities, including those occurring in FA, can be affected by epigenetic changes [24,25].

Vitamin D_3_ is an unique molecule, since its most biologically active metabolite, 1,25(OH)_2_D_3_, acts as a high affinity ligand of the transcription factor VDR [26]. Moreover, vitamin D_3_ is inexpensive, readily accessible and has a favorable side effect profile [27]. Vitamin D has a direct effect on gene regulation, since VDR induces changes to the epigenome of healthy and neoplastic cells influencing their transcriptome [28] (Figure 1). Thus, 1,25(OH)_2_D_3_ is an epigenome modulating signaling molecule [29].

The human genome comprises more than 10,000 loci where ligand-activated VDR binds and affects the transcription of more than 1000 genes, which are referred to as vitamin D target genes [30]. Importantly, a number of these target genes are involved in the modulation of the cells of the immune system, in particular of innate immunity [31], including their growth, differentiation and apoptosis [32]. This suggests that vitamin D and its receptor induce epigenetic programing of myeloid cells during immune challenges, such as infections, tissue damage and by pre-neoplastic cells.

In this review, we discuss the changes in the epigenome, in particular of immune cells, which are of importance for the clinical manifestation of FA. These epigenetic changes can be modulated by vitamin D suggesting that the individual’s vitamin D status and the personal vitamin D response index of FA patients is of critical importance for the progression of the disease.

## 2. Clinical Features of FA

FA was described first in 1927 by the Swiss pediatrician Guido Fanconi [33]. Clinically the disease is often summarized with the triad of congenital malformations, a progressive bone marrow failure and a dramatically increased risk of developing cancer [34]. This rare inherited multisystem disorder is mostly associated with a broad clinical spectrum consisting of typical malformations but can show in some cases also a completely normal phenotype [2,35]. FA-typical malformations present mainly in the skeletal system, especially at the radius/thumb [36]. Moreover, FA frequently affects the whole body, such as low body weight and reduced height, but also malformations in the skin (café au lait spots) [37] or in inner organs, such as heart, kidney and intestine, are common features of the disease [38]. In addition, approximately 80% of all FA patients show signs of an ineffective hematopoietic system and develop bone marrow failure, myelodysplastic syndrome or acute myeloid leukemia [4,34]. Declining blood counts affecting all blood lineages are often the first sign of these hematological features and commonly present already in the first decade of life [39]. Like in other syndromes associated with bone marrow failure, the myeloid system is more severely affected than the lymphoid system, which provides the patients with a high risk of acute infections.

In the past, progressive bone marrow failure was the main cause of death of FA patients [40]. Hematopoietic stem cell transplant is the only curative treatment for the hematological complications. Improved outcome of these transplants are the main reason why today’s FA patients have a higher life expectancy [41]. This is mainly due to higher donor availability, individual treatment protocols [42] and more advanced therapies against graft-*versus*-host disease and viral infections [43,44]. Moreover, better acute myeloid leukemia and myelodysplastic syndrome surveillance, such as frequent checks for chromosomal changes [45], drastically improved the identification of FA individuals at risk. At present, a treatment with synthetic testosterone analogs at supra-pharmacological doses accomplishes stabilization and increase of declining blood counts [46,47,48,49] but cannot prevent myelodysplastic syndrome or acute myeloid leukemia. However, mechanistically this treatment of the hematopoietic system is not well understood [50] and side effects can be therapy limiting [51].

Individuals with FA carry an enormous risk of developing squamous cell carcinoma, especially of the oral mucosa but also in the pharynx, larynx, esophagus, anus and vulva. Compared with the average population these cancers arise at much earlier age and there is a tendency for frequent syn- and meta-chronic squamous cell carcinomas [52]. Due to the genetic defect underlying the disease, treatment options are mostly limited to surgical removal of the cancer [53,54]. Thus, at present squamous cell carcinomas are the most life-threatening complications for adult FA patients. Despite the clinical significance of squamous cell carcinomas, there is still a lack of knowledge as to why FA patients have such an elevated risk of this type of cancer, which is rather uncommon in the general population. Premature aging, DNA fragility, endogenous and exogenous exposure to aldehydes, infections with the human papillomavirus and other chronic infections or inflammations have been discussed in this context but a clear mechanistic explanation is still lacking [55,56,57,58,59,60,61,62].

In addition to these life-threatening and limiting complications, an FA individual is facing numerous other clinical dysfunctions. FA patients have a wide range of metabolic and endocrine impairments [63,64] affecting lipid metabolism [65], glucose and/or insulin homeostasis [66], the thyroid axis [67] and most important fertility [68]. These non-cancerous aspects of FA often dramatically reduce the quality of life of the individuals.

## 3. Genetic and Molecular Features of FA

Until now, 23 genes have been identified that associate with FA, in the majority of which the mode of inheritance is autosomal recessive [69]. Patients with the complementation group R (*FANCR*) carry a heterozygous mutation in the RAD51 recombinase (*RAD51*) gene [69,70], whereas the *FANCB* gene is located on the X chromosome [71]. The main cellular function of FA genes is maintaining genomic integrity during DNA replication via intra-strand cross-linking repair and controlling the replication fork [3]. FA proteins are linked to homologous recombination conducting DNA repair; in the canonical pathway the so-called upstream FA core complex proteins activate the FANCI-FANCD2 complex via mono-ubiquitination [72], which promotes recruitment of DNA repair effectors to chromatin lesions, in order to resolve DNA damage and mitosis. Some of these downstream FA genes are known as tumor suppressor genes in other monoallelic inherited cancers like breast and ovarian cancer (*FANCD1 = BRCA2, FANCS* = *BRCA1, FANCN* = *PABLB2, FANCJ* = *BRIP1, FANCO* = *RAD51C*). Impairment in the FA pathway leads to increased spontaneous and inducible chromosomal fragility [73] and cell cycle arrest [74], which are both hallmarks of the cellular phenotype of FA.

In recent years, the molecular understanding of the role of FA proteins has rapidly grown in addition to functions in genomic maintenance and homeostasis mainly during replication. For example, FA proteins are linked to aldehyde detoxification [75,76] and altered selective autophagy, a key step in immunity, leading to increased mitochondrial reactive oxygen species-dependent inflammasome activation and mitophagy [77]. Moreover, altered mitochondrial functions [78,79,80] and increased oxidative stress [81,82] are linked to FA. This also implies direct interaction of FA proteins with altered insulin secretion [66] and lipid metabolism [83]. Furthermore, non-canonical functions of FA proteins in the control of cytokines, such as tumor necrosis factor [84] and transforming growth factor beta [85] have been described.

Taken together, the understanding of FA as a pure DNA damage repair disease shifts towards a more holistic view, shedding light on energy metabolism. Thus, the cellular and the clinical phenotype of FA can also be subsumed as a premature aging syndrome [60,86].

## 4. FA and Cancer

**Figure 2:**
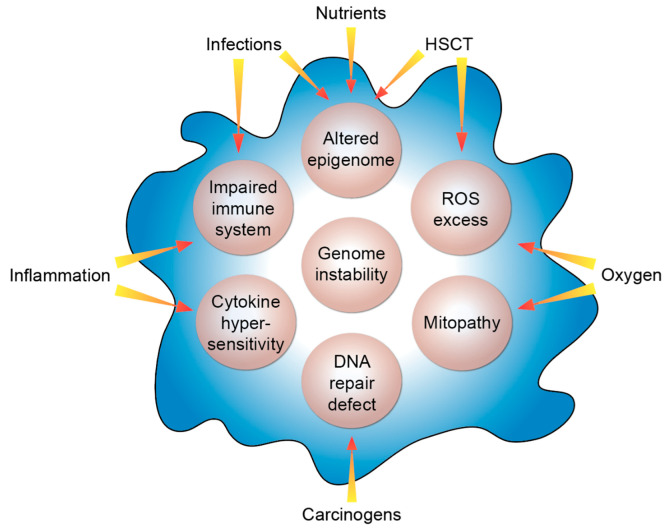
Molecular features of FA. The listed intrinsic processes (beige circles) are modulated by the indicated extrinsic factors. HSCT, hematopoietic stem cell transplant; ROS, reactive oxygen species.

Based on its clinical and cellular phenotype FA can also serve as a cellular model for the study of general molecular functions and physiological aspects like aging as well as other non-communicable diseases occurring in the general population. In that respect, the study of FA had a considerable impact on the molecular understanding of breast/ovarian cancer [87]. Moreover, FA genes are also frequently mutated or dysregulated in sporadic cancers [88] as well as in childhood cancers [89]. Nevertheless, the enormous cancer risk of FA patients still needs to be elucidated mechanistically. Herein, the disturbances of the different FA genes represent the key intrinsic factors of the fragile system of FA individuals (Figure 2). 

The different clinical presentations of FA patients point out that a number of extrinsic modifiers have a significant modulatory impact on the course of the disease. Like in tumorigenesis, the disease modifiers do not need to be disease causing by themselves. Typical examples of such extrinsic factors can be oxygen, inflammation or infections like by human papillomavirus or *Candida albicans*. Additionally, carcinogens at low concentrations can tip the scale. Another example is hematopoietic stem cell transplant: FA patients exhibit an up to 700-fold increased risk for the development of squamous cell carcinomas compared to the general population [4] but in FA individuals that had received a hematopoietic stem cell transplant, this risk is even more elevated and squamous cell carcinomas occur in them at a younger age [5,90]. Risk analyses have identified graft-*versus*-host disease, i.e., a dysregulation of the transplanted immune system of the donor, as the underlying factor [91]. In balance of these negative extrinsic factors, nutrients, such as vitamin D, can act as positive extrinsic modifiers mainly via affecting the epigenome. Thus, intrinsic and extrinsic factors together determine the clinical individual cause of the disease. 

Despite its rareness, the hematopoietic clonal disease and expansion in FA are intensely studied. Somatic amplifications at chromosomes 1q, 3q (including the gene *MECOM* (MDS1 and EVI1 complex locus)), and deletions of chromosome 7 during the aplastic phase of the disease display the origin of the respective clones [45,92,93,94]. In that respect, *MECOM* plays a crucial role as it encodes for a transcriptional regulator with an essential role in hematopoiesis and mediating epigenetic modifications by interacting with DNA, proteins and protein complexes [95]. Thus, the overexpression of *MECOM* provides the cell with growth advantages and disturbs the epigenetic landscape. Moreover, at the stage of myelodysplasia, de-regulations of the *RUNX1* gene are frequently found [93]. Thus, the clonal expansion of such altered hematopoietic cells ultimately leads to myelodysplastic syndrome and acute myeloid leukemia [92]. Furthermore, in FA the changes on chromosomes 1, 3 and 7 are associated with a negative outcome after hematopoietic stem cell transplant [45]. Even though the association between these specific chromosomal changes and disease progression towards acute myeloid leukemia is well characterized, it is still not elucidated why and how those initial changes arise.

Naturally, studying negative disease modifiers is much easier than identifying and attributing the significance of preventive modifiers, such as vitamin D. Therefore, there is still a lack of knowledge in determining specific preventive factors besides a general healthy lifestyle, e.g., physical activity, healthy diet and the avoidance of smoking [96]. In summary, the occurrence of inherited mutated FA genes primarily indicates the fragility of the system “health”, while intrinsic and extrinsic factors are the real modifiers of the disease. As FA gene mutations cannot be changed in the whole body, the modulation of disease modifiers bears the potential of therapy and even disease prevention.

## 5. The Impact of Epigenetics in FA

The protein-DNA complex of histones and genomic DNA is referred to as chromatin [7]. The key function of chromatin is to keep most of the genome inaccessible to transcription factors and RNA polymerases, i.e., in a cell- and tissue-specific fashion chromatin functions as a gatekeeper for undesired gene activation. Differentiation processes are controlled by epigenetic programing, i.e., a change of the so-called epigenetic landscape composed of transcription factor binding, histone modifications and chromatin accessibility [97]. Thus, through epigenetics terminally differentiated cells have a permanent memory about their identity [15].

Next-generation sequencing techniques, which had been developed after the sequencing of the human genome, such as chromatin immunoprecipitation sequencing (ChIP-seq) and formaldehyde-assisted isolation of regulatory elements sequencing (FAIRE-seq), allow the genome-wide assessment of the transcription factor binding, histone modifications and chromatin accessibility [104]. These approaches have been systematically applied by large research consortia, such as ENCODE (www.encodeproject.org) and Roadmap Epigenomics (www.roadmapepigenomics.org), for the epigenome-wide characterization of more than one hundred human cell lines [105] and a comparable number of primary human tissues and cell types [106], respectively. It should be kept in mind that every single cell of an individual carries the same genome, but that there are hundreds to thousands different epigenomes, in which the tissues and cell types differ significantly.

**Figure 3:**
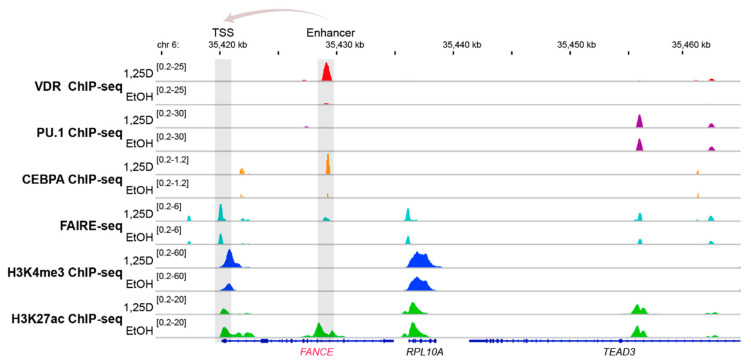
Vitamin D-triggered epigenomic profile in the region of the *FANCE* gene. The IGV browser [98] was used to display the epigenomic profiles at enhancer and transcription start site (TSS) regions of the vitamin D target gene *FANCE*. THP-1 cells had been treated for 24 h with 1,25(OH)_2_D_3_ (1,25D) or vehicle (EtOH) and in three biological repeats, ChIP-seq experiments had been performed with antibodies against VDR [99], the pioneer factors PU.1 [100] and CEBPA [101], the histone marker for active transcription start site (TSS) regions, H3K4me3 [102] and the marker for active chromatin, H3K27ac [102], such as at enhancers, as well as FAIRE-seq [103] for accessible chromatin. The gene structures are shown in blue and vitamin D target gene *FANCE* is indicated in red. The genes *RPL10A* and *TEAD3* serve as non-regulated references.

The genomic region of the vitamin D target gene *FANCE* [103] serves as an illustrative example of vitamin D-triggered epigenetic changes in the context of FA (Figure 3). The *FANCE* gene encodes for a critical protein of the FA core complex mediating FANCD2/FANCI mono-ubiquitination, which is the essential activation step of the FA/breast cancer DNA-repair pathway [107]. In the monocytic cell line THP-1, which was derived from a 1-year old male patient with acute myeloid leukemia [108], ChIP-seq indicated a VDR binding site 9 kb downstream of the transcription start site of the *FANCE* gene. Within this enhancer region 1,25(OH)_2_D_3_ not only significantly increased the binding of VDR but also of its pioneer factor CEBPA. In parallel, at this genomic region the amount of accessible chromatin as well as the histone marker of active chromatin, H3K27ac, raised after treatment of the cells with the VDR ligand. Looping of this enhancer to the transcription start site of the *FANCE* gene results in 1,25(OH)_2_D_3_-triggered changes of accessible chromatin, H3K27ac markers and markers of active transcription start sites, H3K4me3. Taken together, vitamin D changes specifically on the level of VDR and CEBPA binding, chromatin markers and accessible chromatin of the epigenome at the region of the *FANCE* gene.

In general, epigenetics associates with lifestyle and environmental conditions of healthy as well as of diseased individuals, such as FA patients [109]. The dynamic profile of the epigenome provides the advantage that some events of epigenetic programing are reversible. This implies that lifestyle changes can improve health and prevent or milden disease, such as complications of FA. Thus, as long as no irreversible tissue damage has happened, it is in the hands of the individual to reverse a disease condition. Accordingly, there is a high level of individual responsibility for staying healthy and epigenetics provides a molecular explanation for this life philosophy [15].

## 6. Vitamin D, Immunity and Cancer

Vitamin D_3_ is produced from 7-dehydrocholesterol in a non-enzymatic reaction that requires the energy of UV-B (290–315 nm) radiation [110]. Humans are able to synthesize the molecule themselves in the upper layers of their skin, i.e., the term “vitamin” may not be correctly used. However, most individuals have low endogenous vitamin D_3_ production, since they stay most of the day indoors, cover their skin by textiles outdoors and/or use sunscreen in order to prevent sunburn. Since average human diet is low in vitamin D_3_ sources, such as the fatty fish tuna, sardines, salmon and mackerel, worldwide more than a billion persons are vitamin D deficient [111]. Thus, direct supplementation with vitamin D_3_ is recommended [112].

**Figure 4:**
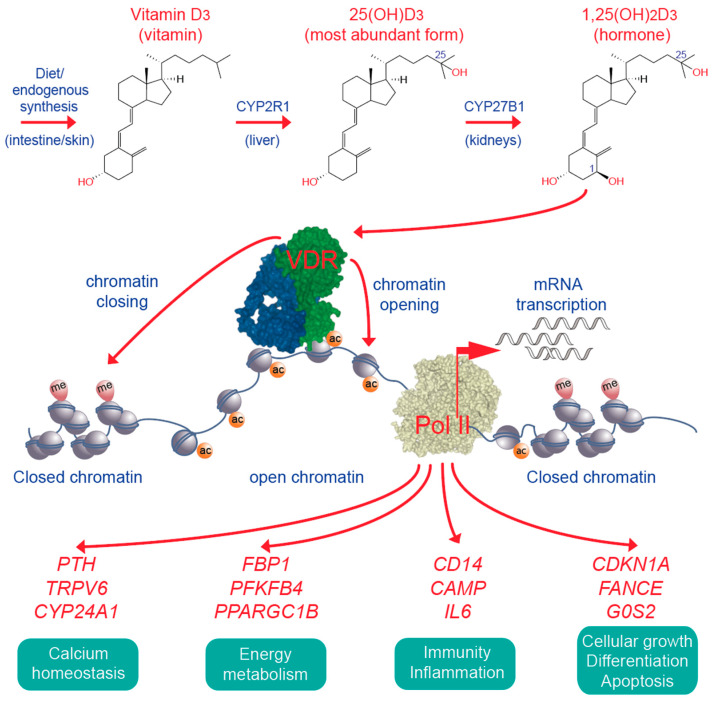
Key elements of vitamin D signaling. Vitamin D_3_ is obtained from diet or supplementation or synthesized endogenously in UV-B exposed skin. In the liver and the kidneys vitamin D_3_ is converted to 25(OH)D_3_ and 1,25(OH)_2_D_3_ (top). The latter activates the VDR (green), which bind together with its preferred co-receptor retinoid X receptor (blue) to accessible genomic binding sites (center). In the chromatin environment of its binding sites activated VDR affects chromatin opening and closing (see Figure 3 as illustrative example), which results in the up- and down-regulation of vitamin D target genes. Representative vitamin D target genes are sorted into four different physiological functions (bottom). Pol II, RNA polymerase II; CYP, cytochrome P450.

In the liver, vitamin D_3_ is converted to 25-hydroxyvitamin D_3_ (25(OH)D_3_), the serum concentration of which is used as a marker for the vitamin D status of an individual [113] (Figure 4). Although debated [114,115], there is rather general consensus that the 25(OH)D_3_ serum levels should be in the range of 75–150 nM, i.e., 30–60 ng/mL, in order to achieve benefits form vitamin D [116]. In the kidneys as well as in some epithelial and immune cells, a small amount of the circulating 25(OH)D_3_ is hydroxylated further to 1,25(OH)_2_D_3_, which acts as the high-affinity (K_D_ = 0.1 nM) VDR ligand [117]. The transcription factor VDR is one of 48 members of the nuclear receptor superfamily, prominent representatives of which are the receptors for cortisol, estrogen and testosterone [118]. VDR is the only protein that binds 1,25(OH)_2_D_3_ at sub-nanomolar concentrations [119], i.e., the transcription factor mediates all effects of physiological vitamin D concentrations.

Full 1,25(OH)_2_D_3_-sensitive VDR evolved first some 550 million years ago in a boneless fish [120,121]. In analogy to the evolutionary history of related nuclear receptors [122,123], the prime function of vitamin D and its receptor VDR is assumed to be the control of energy homeostasis. A substantial amount of the energy was needed by the innate immune system and the emerging adaptive immune system [124]. VDR obtained a modulatory role on immunity via the control of immunometabolism [125]. Importantly, for a proper function of the immune system, its cells need to grow very rapidly. Therefore, the functions of vitamin D and its receptor spread also to the control of cellular proliferation, differentiation and apoptosis [126]. When some 400 million years ago some fish species left the calcium-rich ocean and populated the calcium-poor land, VDR took the additional function of regulating calcium homeostasis [127]. The latter is essential for bone formation and evolved to a physiological function, in which vitamin D became indispensable [128]. For this reason the dominating phenotype of vitamin D deficiency is bone malformation, such as in rickets [129].

The most important extra-skeletal function of vitamin D is the modulation of the immune system, such as stimulating the innate immune system in its fight against bacterial infections [130] and preventing autoimmune diseases that may be caused by overreactions of the adaptive immune system [131,132]. This modulatory role of vitamin D actions involves the control of growth, differentiation, activation/deactivation and eventually apoptosis of monocytes, dendritic cells and different types of T cells [133] (Figure 4). Together with the pioneer factors CEBPA and PU.1 VDR is of key importance for the differentiation of monocytes and granulocytes [134], possibly via the vitamin D target gene *CDKN1A* encoding for a cyclin-dependent kinase inhibitor [135,136]. Additionally, vitamin D regulates the number of embryonal hematopoietic stem cells [126]. Vitamin D deficiency was also shown to be associated with increased complications, including graft-*versus*-host disease, in the context of hematopoietic stem cell transplants [137]. Moreover, VDR was identified as a new genetic modifier contributing to the expression of different subtypes or acute myeloid leukemia [138]. Thus, vitamin D plays an important role in normal but also in pathological hematopoiesis.

Vitamin D potentiates the activity of cells of innate immunity, such as monocytes, macrophages and natural killer cells, while it represses T_H_ (T helper) 1 cells of adaptive immunity and increases the number of T_H_2 cells and T_reg_ (T regulatory) cells. It is important to note, that rapidly growing immune and cancer cells use the same signal transduction pathways and genes for controlling their proliferation, differentiation and apoptosis [139]. Thus, the anti-cancer potential of vitamin D on cancer cells can be understood as a side effect of managing the immune system, such as modulating immune cells of the tumor microenvironment [140,141]. Nevertheless, controlling and reducing settled tumors is probably not the main anti-cancer effect of vitamin D. The key function of VDR and its ligand probably is the prevention of tumor establishment. In every individual per day thousands of cells transform from a normal to a malignant phenotype, but fortunately they are detected and destroyed by cytolytic T cells [142]. Thus, vitamin D is most effective in preventing cancer onset by increasing the infiltration of CD8^+^ cytolytic T cells with an active effector memory phenotype into the tumor site [143]. This important change of the tumor microenvironment is based on the anti-inflammatory action of vitamin D via decreasing the expression of the pro-inflammatory cytokine IL6.

## 7. Impact of Vitamin D Status and Response Index

Seasonal changes of sun exposure at higher latitudes, such as in Europe, cause over the year variations in the vitamin D status of non-supplemented individuals [144]. This led to discussions on the minimal suggested vitamin D status, which is based on the US Institute of Medicine [114] a serum 25(OH)D_3_ concentration of 50 nM, while at least 75 nM are suggested by the US Endocrine Society [145]. Accordingly, there are different recommendations for daily vitamin D_3_ supplementation ranging from 800 to 4000 IU/day (20–100 µg). However, probably not all individuals have the same needs for vitamin D_3_, i.e., it is questionable, whether a threshold level of the vitamin D status is an optimal reference. Nevertheless, patients with hematological diseases often have a low vitamin D status, which is associated with an unfavorable prognosis [146,147,148,149].

**Figure 5:**
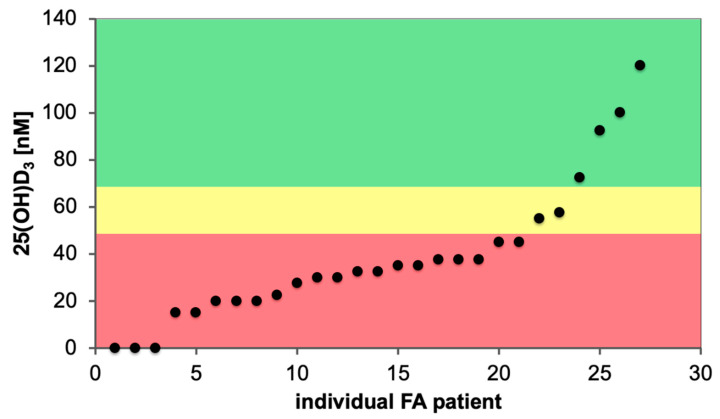
Vitamin D status of FA patients. The serum 25(OH)D_3_ concentration of 27 randomly chosen FA patients of a single center are displayed, 21 of which are vitamin D deficient (red), while 2 have an insufficient (yellow) and 4 a sufficient (green) vitamin D status.

We analyzed the vitamin D status of newly diagnosed FA patients in a single center and observed that the majority of them were vitamin D deficient (Figure 5). FA cohorts described in the literature report similar percentages (70%) of individuals as vitamin D insufficient [63,67]. This represents a far higher rate of vitamin D deficiency as in the general population [111,150]. FA patients display stem cell exhaustion and an increased risk of developing myelodysplastic syndrome or acute myeloid leukemia. This suggests a detrimental effect of vitamin D deficiency in this life-limiting disease.

Therefore, the general recommendation for FA patients is an annual measurement of their vitamin D status and an appropriate supplementation [64]. Moreover, in view of the biological effect of vitamin D on the epigenome, a normalization of the vitamin D status also might bear prevention potential for FA disease progression and FA related complications. So far, no clinical trials have been conducted investigating this “preventive hypothesis” in FA, but in analogy to other chronic diseases it is very likely [151]. Anecdotal personal observations (E. Velleuer) support this assumption as the normalization of the individual vitamin D status in FA patients goes along with an improved homeostasis, e.g., the decrease of elevated liver counts, the reduction of cardiac iron levels in non-transfused FA patients as well as the stabilization of blood counts.

Based on the concept of the vitamin D response index, persons are distinguished into high, mid and low responders to vitamin D [152]. Thus, every individual has a personal need for vitamin D_3_ supplementation, which depends on the relation of vitamin D status and response index. The index is based on the molecular response to vitamin D and is determined via the change in the expression of vitamin D target genes after a significant change in the vitamin D status [153]. Thus, the vitamin D response index is an (epi)genetic trait that is constant over time or may change only slowly during the development of an age-related disease. High vitamin D responders have a functional vitamin D system already at a low vitamin D status and are better prepared to tolerate conditions of low or no endogenous vitamin D_3_ production, such as during European winters. Accordingly, they should be less affected by infections [154], autoimmune diseases [155] and cancer [32], against which vitamin D has a protective function. In contrast, low vitamin D responders have to reach a higher vitamin D status by higher daily supplementation (maybe 50–100 µg/day), in order to obtain the full benefit of vitamin D. Based on the vitamin D_3_ supplementation studies VitDmet (NCT01479933, ClinicalTrials.gov) [156] and VitDbol (NCT02063334) [157] some 25% of the individuals are low responders. It is assumed, that this percentage also applies to FA patients.

In summary, the vitamin D response index may be more suited for tailoring the daily vitamin D supplementation needs of healthy and diseased individuals than referring exclusively to the vitamin D status. FA patients need special attention and mostly a daily supplementation of 1 µg (40 IU) vitamin D_3_/kg body weight especially during winter is needed for achieving normal vitamin D levels, i.e., a 50 kg person should take 2000 IU per day (E. Velleuer, own observations).

## 8. Limitations and Future Directions

This review aimed to bring together two different fields, FA and vitamin D, that had not been jointly presented before. We used epigenetics and the immune system as a bridge between both fields, but we admit that far more research needs to be done before we can fully appreciate the impact of vitamin D_3_ for FA patients. Because FA is a rare disease, large randomized controlled trials are not feasible. Nevertheless, smaller scale vitamin D intervention studies with FA patients will be very instructive, in particular, if they involve transcriptome- and epigenome-wide analysis. This may widen the perspective on FA in regard to its epigenetic component, which is essential for the personalized treatment of the disorder.

## 9. Conclusions

FA is well understood as a collection of at least 23 different monogenetic diseases affecting the DNA double-strand repair system. However, the onset of the outbreak of severe consequences of the disease are very individual and seem to depend on the lifestyle and environment of the FA patient. Lifestyle decisions, such as composition of diet and the exposure to carcinogens like tobacco smoke, as well as environmental encounters, such as virus and bacterial infections, trigger signal transduction cascades that often result in transient and persistent changes of the epigenome. The nature of its function makes the immune system a special target of epigenetic changes. Thus, the dynamic epigenome can have beneficial as well as detrimental effects on the progression of FA.

Vitamin D is a safe natural compound with an epigenome modulating function [29]. The epigenome-wide activity of vitamin D is essential for understanding the physiological impact of the nuclear hormone, in particular on immune cells, such as monocytes and their differentiated subtypes. Accordingly, vitamin D_3_ supplementation, ideally being personalized based on individual’s vitamin D response index, is assumed to enable proper epigenetic programing of immune cells throughout hematopoiesis as well as during antigen encounter. In this way, sufficient vitamin D levels should contribute to the maintenance of wellbeing and the prevention of the onset of diseases and disease progression, especially in genetic disorders like FA.
